# Municipal Milk- and Meat-Inspection1Read before the Missouri Valley Veterinary Association, December 11, 1899.

**Published:** 1900-02

**Authors:** E. J. Netherton

**Affiliations:** St. Joseph, Mo.


### MUNICIPAL MILK- AND MEAT-INSPECTION.[1]

By E. J. Netherton, V.S.,
St. Joseph, Mo.

The production and distribution of milk- and meat-products engage the attention of no small part of our population. The use of milk is general and not limited to any class or locality. It is regarded as a necessity by almost every family, and for this reason information regarding it is important. By milk is meant the properly handled product of healthy, well-fed cows. It is a little heavier than water. Its specific gravity varies from 1029 to 1033. The first milk given after birth is called colostrum. It contains a large proportion of albuminoids, and is not fit for food except for the new-born, and any milk having a large amount of sediment is suspicious.

[1] Read before the Missouri Valley Veterinary Association, December 11, 1899.

Particles of dirt are signs that germs are abundant. Thus dirty milk may be dangerous as well as disgusting. The dirt in milk consists mostly of particles of dead skin and manure which fall into the pail from the body of the cow during milking, but dirt and dust in the vessels used for handling milk and unclean attendants are also common sources of dirty sediment in milk.

Milk from unhealthy or unthrifty cows, or that which has been handled by sick persons, is dangerous, as it contains infectious germs or foreign substances which may affect the health of the consumer. The germs of diphtheria, typhoid and scarlet fever and tuberculosis (consumption) have been found in milk, and thus transmitted to man and spread from family to family. Feverish cows, those having just given birth to young, and sometimes cows that have been milked a long time, produce milk which should not be used. Any milk having an unnatural appearance or odor should be discarded.

The most common forms of fraud practised with milk are the removal of part of the cream by the aid of a milk-separator and the addition of water. Most communities have laws against adulteration, but they are not as rigidly enforced as they should be. The law in this State requires at least 3 per cent. of butter fat, but the custom among dairymen is to have their milk come barely within the bounds of the law, and no more. If a dairyman is dishonest enough to water his milk he will not be careful about the purity of the water added. Contagious diseases have been traced directly to contaminated water added to milk for the purpose of adulteration or which has been used to rinse the cans. There are also various chemicals added as preservatives. The most common substances contain salicylic acid, boric acid, borax, or formaldehyde. These are not regarded as poisons, but when taken regularly in small doses in milk they have an injurious effect upon the system.

But here is not where the danger lies. The chief object of inspection is to determine whether the meat- and milk-products are from animals that are free from diseases, especially tuberculosis. It has been known for some years that the milk of cows suffering from tuberculosis may contain tubercle bacilli. The bacilli consumed in the milk by infants and children may lead to tuberculosis (consumption) in one or more of its numerous forms. It has been the object of investigators, both in the medical and veterinary professions, to find out exactly under what condition the milk from tuberculous cows may be dangerous. Both classes maintain that the tubercle bacilli may pass into the milk when a cow is affected with tuberculosis to a marked degree or even when the udder remains free from disease. Tuberculous deposits in the udder, when they have reached a certain size, can be detected in life. For this reason the inspector should condemn the milk of all cattle suffering from tuberculosis, because even if the udder is not visibly diseased tuberculosis may nevertheless be present. The great importance of a regular periodical inspection of dairy cattle is thus made manifest. If only such animals in which the udder is found diseased would be condemned and the milk rejected a large amount of the injury done by tubercle bacilli in milk could thereby be avoided. Those tuberculous animals in which the disease of the udder escapes attention because of its restricted character would still distribute tubercle bacilli. But inspection done by a competent veterinarian would largely relieve apprehensions of the public which have been aroused by the extended discussion which this dread plague of tuberculosis has undergone in all journals. But the public should be made aware of the great danger in the use of milk from cows which are beginning to emaciate or whose udders are diseased. If we bear in mind the wide distribution of infectious diseases in cattle, especially dairy herds, it would lead us to believe that dairy herds collected in different localities were always found to be more or less diseased or infected. It is essential, therefore, to guard the milk-consuming public against disease, to look with suspicion upon all dairy cattle, and more especially those that have not been inspected. The greater dependence of the cities on the food furnished in the shape of meat and milk has with unerring certainty multiplied the number of tuberculous cases. If mothers better appreciated the dangers to which their little ones are exposed, numerous diseases occasioned by the use of meat from animals teeming with bacterial growth, more of them would exercise their influence in securing the proper inspection of these products. It behooves the city officials and the veterinarians as sanitarians to exercise their most energetic influence in awakening the minds of the people to the dangers thus lurking in the meat- and milk-supply, and from the dangerous power that the milk-supply of our city exercises in acting as the vehicle of transportation of such diseases as tuberculosis, diphtheria, typhoid and scarlet fever, and many others.

Under the present existing circumstances we have slaughtering establishments owned by butchers who buy their stock for slaughter where they choose, and they are beyond the reach of the Federal authorities. Why? Because government inspection only applies to abattoirs having an interstate trade. These parties say they are only supplying the local and State trade, and, therefore, are at perfect liberty to buy and slaughter any kind of stock they choose, be it healthy, crippled, or diseased. Here State and municipal laws come into control, if any law exists. These places have been visited by the writer, and the unspeakable filth and lack of all cleanliness point them out as sources of disease and a standing menace to the consumer of meats slaughtered there, without consideration of class of animals killed. It is true there are exceptions to the rule, but they are very few. If any adequate reason ever existed for Federal meat-inspection the same reasons are of equal force as applying to State and municipal inspection. For this inspection to be complete a man who is a graduate veterinarian should have charge of the inspection of all animals killed or offered for slaughter, and the stamp or official seal of soundness should be affixed to his products, so it will be free from all suspicion of disease and stand equally as good before the public as meats from abattoirs licensed by the United States authorities. It is said no law is stronger than the public opinion behind it. I feel confident public opinion in this city will be forceful enough to sustain all municipal legislation on this subject. Next, the veterinarian appointed to enforce the ordinance should be a member of the board of health, for it certainly is a matter concerning the health of many people. He thus has representative men to reinforce his decisions. Again, it is not a question of politics, but of efficiency, and should be free from politics. He should receive a salary commensurate with his services, and should devote his time to the work along with other duties that will properly come within his province. The municipal control of meat- and milk-supply in cities is one of the necessities of the times if we wish to keep disease from doing its deadly work.

That milk is a vehicle by which disease is transmitted is a well-established fact in the minds of all educated men. In how many ways an animal may be the source of infection it would require too long a time to describe. But tuberculosis and other diseases of the udder and teats suggest abundant causes of infection. The determination of these causes can only be described by the veterinarian. That diseased and tuberculous cows are in our herds is true if the observations and experience of other cities are true. The municipality, the council, or board of health have the undoubted right to take the whole subject of meat- and milk-inspection under city control, and it is for them to enact ordinances protecting the public from diseased meat and impure milk. The salary of an educated and competent veterinarian would approximately be $1200. Place the meat and dairy inspection under his control as a city official, and license all butchers and dairymen, but hold them to a strict conformity to the ordinance, or revoke their license and publish the facts. To neglect wise and thorough control of the meat- and milk-supply of any city is to invite the dissemination of diseases of the most prevalent and fatal character. The municipal authority of this city should stand for the health of this community, for the health of the babies and invalids who depend upon this milk for food. Are the people willing to take the risk? I am of the opinion that the lack of such inspection and control is due not to the wilfulness, but to indifference and ignorance of the true conditions. Let the people agitate until their will is obeyed.

